# Endocrine therapy initiation, discontinuation and adherence and breast imaging among 21-gene recurrence score assay-eligible women under age 65

**DOI:** 10.1186/s13058-017-0837-2

**Published:** 2017-03-31

**Authors:** Suzanne C. O’Neill, Claudine Isaacs, Filipa Lynce, Deena Mary Atieh Graham, Calvin Chao, Vanessa B. Sheppard, Yingjun Zhou, Chunfu Liu, Nandini Selvam, Marc D. Schwartz, Arnold L. Potosky

**Affiliations:** 1 Georgetown Lombardi Comprehensive Cancer Center, 3300 Whitehaven Street, NW, Suite 4100, Washington, DC, 20007 USA; 2 Hackensack Meridian Health, Hackensack, NJ USA; 3 Genomic Health, Inc, Redwood City, CA USA; 4 School of Medicine Virginia Commonwealth University, Richmond, VA USA; 5 HealthCore, Inc, Wilmington, DE USA; 6 IMS Health, Fairfax, VA USA

**Keywords:** Breast cancer, Gene expression profiling, Genomic testing, Endocrine therapy, Breast imaging

## Abstract

**Background:**

Aside from chemotherapy utilization, limited data are available on the relationship between gene expression profiling (GEP) testing and breast cancer care. We assessed the relationship between GEP testing and additional variables and the outcomes of endocrine therapy initiation, discontinuation and adherence, and breast imaging exams in women under age 65 years.

**Methods:**

Data from five state cancer registries were linked with claims data and GEP results. We assessed variables associated with survivorship care outcomes in an incident cohort of 5014 commercially insured women under age 65 years, newly diagnosed with stage I or II hormone-receptor-positive, human epidermal growth factor receptor 2 (HER2) non-positive breast cancer from 2006 to 2010.

**Results:**

Among tested women, those with high Onco*type* DX® Breast Recurrence Score® (RS) were significantly less likely to initiate endocrine therapy than women with low RS tumors (OR 0.40 (95% CI 0.20 to 0.81); *P* = 0.01). Among all test-eligible women, receipt of Onco*type* DX testing was associated with a greater likelihood of endocrine therapy initiation (OR 2.48 (95% CI 2.03 to 3.04); *P <*0.0001). The odds of initiation were also significantly higher for tested vs. untested women among women who did not initiate chemotherapy within six months of diagnosis (OR 3.25 (95% CI 2.53 to 4.16)), with no effect in women who received chemotherapy. Discontinuation and adherence and breast imaging exams were unrelated to tested status or RS.

**Conclusions:**

Lower endocrine therapy initiation rates among women with high RS tumors and among untested women not receiving chemotherapy are concerning, given its established efficacy. Additional research is needed to suggest mechanisms to close this gap.

## Background

Women with estrogen-receptor-positive (ER+), early-stage disease represent more than half of the almost 250,000 women newly diagnosed with breast cancer each year [1–4]. Clinical guidelines for these women now include results from genomic expression profiling (GEP) tests, such as the Onco*type* DX® Breast Recurrence Score® (RS) [5–7], to refine recurrence risk estimates and guide treatment selection. These guidelines have led to broad dissemination and integration of such tests into routine oncology care [8–10]. In the absence of genomic profiling, combined chemo-endocrine therapy is recommended for most of these patients [11]. However, in the presence of a low RS, patients who receive this result (almost 50% of patients) can receive endocrine therapy alone, safely forgoing chemotherapy and its side effects [6]. Approximately 25% of patients receive high-risk results and can be prompted to act on this risk and receive chemotherapy to maximize survival [12]. Previous studies show that receipt of chemotherapy generally follows these recommendations, with some observed variations [9, 13, 14].

There have been no studies of other possible and unintended effects of GEP testing on breast cancer care. For example, we do not know whether survivorship care, including both endocrine therapy and breast imaging for recurrence or progression, received by this growing group of breast cancer survivors is affected by GEP testing. All women eligible for Onco*type* DX testing should also receive endocrine therapy over at least a 5-year period after the conclusion of initial surgery (and chemotherapy or radiotherapy if given) [7]. It is estimated that approximately 10–30% of all women with hormone-receptor-positive breast cancer do not initiate endocrine therapy [15–17] and 40–60% may not complete the full 5-year regimen of therapy [18–21]. Likewise, 21% of all women with early-stage breast cancer do not receive breast imaging tests for recurrence in the year following diagnosis [22], even though practice guidelines recommend an annual follow-up mammogram after initial surgery [23, 24]. Therefore, the objective of this study was to measure the rates of initiation, discontinuation and non-adherence to endocrine therapy and also the receipt of breast imaging (mammography and breast magnetic resonance imaging (MRI)), comparing variations in these care patterns by test result, and differences between GEP-tested and untested women.

## Methods

### Patient selection and study cohort

Details of data linkages have been presented previously [10]. Briefly, our linked database consists of five state cancer registries containing clinical and pathological variables linked with claims data from HealthCore’s Integrated Research Database (HIRD^SM^). HealthCore Inc. (Wilmington, DE, USA) is an independent subsidiary to Anthem, Inc., which is an independent licensee of the Blue Cross and Blue Shield Association. We linked RS results through collaboration with Genomic Health, the patent holder of the Onco*type* DX test.

We conducted a retrospective cohort study of 6737 women who were aged 24–63 years and were diagnosed with stage I or II hormone-receptor-positive human epidermal growth factor receptor 2 (HER2) non-positive breast cancer between 2006 and 2010. We excluded 31 women who initiated endocrine therapy prior to their breast cancer diagnosis, 1179 women who were diagnosed while without medical insurance eligibility and did not initiate endocrine therapy within 6 months from the date of diagnosis, 265 women with two coverage gaps or a gap longer than 6 months or a follow up period shorter than 3 months. Finally because oral medication is the most common form of endocrine therapy, we excluded 248 women who did not have pharmacologic coverage when diagnosed and who had not initiated drug coverage within 4 months of initial diagnosis. Thus, our final cohort for this analysis included 5014 women eligible for GEP testing, who were followed through 30 April 2012 or the end of medical coverage, whichever was earlier. Median follow-up time was 2.68 years. The sample sizes differed in some of the analyses, and this reflects incomplete data on the study variables. Only participants with complete data were retained.

### Study measures

Endocrine therapy initiation was defined as receiving prescriptions for tamoxifen, letrozole, anastrozole, or exemestane within 18 months post breast cancer diagnosis. GEP status and RS were identified by a linkage between HealthCore and Genomic Health test data for Anthem members with breast cancer whose GEP testing is performed by Genomic Health. RS results were categorized as low (RS <18), intermediate (RS = 18–30) or high (RS >30) as defined by the original clinical validation studies for the assay [25]. From registry data we obtained age at diagnosis, race-ethnicity, marital status, date of diagnosis and diagnosis of prior primary cancers other than breast cancer, including non-melanoma skin cancers. Staging was created using the American Joint Committee on Cancer Breast Cancer Staging (version 6 or 7, depending on the diagnosis year) [26].

We obtained estrogen receptor (ER), progesterone receptor (PR), HER2, nodal status and histological grade from registry data. We grouped women with ER+ and PR+ cancer vs. women having either PR+ or ER+ tumors (but not both). We compared borderline HER2 status (immunohistochemical score (IHC) 2+) and those with unknown status to those with HER2-negative cancer. HER2 status was derived using the SEER Collaborative Stage Site-Specific Factor 15 (positive/negative/borderline/unknown). We also compared those with no cancer in the lymph nodes (nodal status (N)0) to those with cancer in the lymph nodes <2 mm in size that can only be seen under a microscope (N1mic) and those with cancer of at least 2 mm in size in at least one of three axillary lymph nodes (N1). Well-differentiated and moderately differentiated tumors were compared to poorly differentiated or undifferentiated tumors. From HIRD we derived 31 comorbid conditions 1 year prior to breast cancer diagnosis based on the Elixhauser comorbidity index [27]. For each condition, we used a commonly applied algorithm that required an inpatient diagnosis and/or at least two outpatient diagnosis codes at least 30 days apart, to minimize false positives. Finally, members’ residential 5-digit zip codes were linked to derive sociodemographic data based on the 2007–2011 American Community Survey of US Census, including median household income (in quintiles) and urban vs. rural location. Chemotherapy was defined as receipt of any guideline-recommended chemotherapeutic agent after surgical resection that was initiated within 6 months of primary surgery.

Receipt of follow-up imaging to assess recurrence or spread of breast cancer was defined using Current Procedural Terminology (CPT)-4 and Healthcare Common Procedure Coding System (HCPCS) codes corresponding to screening mammography, diagnostic mammography, ultrasound imaging or MRI of the breast within the period 6–26 months after initial diagnosis.

### Statistical analysis

We first examined the bivariate relationship between endocrine therapy initiation and each independent predictor variable, using two-sided chi-square and *t* tests as appropriate, to test statistical significance. We then assessed initiation using a multivariable logistic regression model with endocrine therapy initiation within 18 months as the binary dependent variable. One model was run among the entire cohort of those considered eligible for GEP testing to assess the association between GEP testing and endocrine therapy initiation, adjusting for all other variables. Next, a similar model was run among only GEP-tested women to assess the association between RS results (as both categorical and continuous measures) and initiation of endocrine therapy. Prior to running our final multivariable model, we tested several, clinically directed hypothesized interactions (e.g., tested status × receipt of chemotherapy) individually when added to the main effects model. We only report those interaction terms that met our criteria for statistical significance (type I error of 0.05) in the final multivariable model. Models only included women who had complete information on all predictors (93% of all eligible women, 95% of all tested women).

Discontinuation of therapy was defined as having no prescription refill at least 45 days after all pills would be estimated to have been taken: 45 days was chosen because the majority of all prescription fills in our sample (88%) were for a 30-day supply. We conducted multivariate survival analysis using Cox proportional hazard models with competing risks, to identify sociodemographic and clinical variables associated with time to discontinuation. The time of discontinuation was defined as the date of the last fill before the qualified gap, plus the day supply of that fill (usually 30 or 60) plus any pills that remained from previously filled prescriptions.

Competing risk due to disease recurrence or metastatic spread was defined using surrogate variables of inpatient and outpatient claims reflecting either a diagnosis of secondary breast cancer at least 1 year after primary diagnosis or the initiation of a new cycle of chemotherapy following initiation of endocrine therapy. Women were censored at the date of discontinuation of medical and/or pharmacy insurance coverage, or the end of available claims data (30 April 2012), whichever was earlier. We created two Cox proportional hazards models as described previously, comparing tested and untested women with respect to discontinuation, and then the association of GEP test results (RS) with discontinuation.

Non-adherence to therapy was defined as a medication possession ratio (MPR) of <80%, where MPR = the total day’s supply/the total number of days from initiation to the end of follow up, censoring those who had discontinued therapy [18, 28]. Associations between patient-level clinical, sociodemographic, or group-level socioeconomic variables and non-adherence were assessed using a multivariable logistic regression model.

We assessed receipt of at least one breast imaging exam (as defined above) during the period 6–26 months post diagnosis using a logistic regression model similar to the endocrine therapy initiation model. This time range was selected to be as conservative as possible in identifying such exams. We controlled for type of surgery in this model (breast conserving surgery vs. unilateral mastectomy, and exclusion of women who had bilateral mastectomy).

All tests were two-sided and we considered a *P* value <0.05 as significant. We report adjusted odds ratios and 95% confidence intervals (CI) from logistic regression and adjusted hazard ratios with 95% CI for Cox models. All calculations were done using SAS 9.3 (Cary, NC, USA).

## Results

In the study cohort (*N* = 5014), 1607 women (32%) received GEP testing (Table 1). Most of these women had either low (*N* = 821) or intermediate (*N* = 621) risk tumors. The sample was primarily white, previously unaffected by cancer, had no comorbidity and resided in urban areas. Most received breast-conserving surgery as their primary treatment (*N* = 3049).

**Table 1:** Characteristics of selected cohort and relationship with endocrine initiation and adherence

	Initiation			Adherence		
	Selected cohort			Selected cohort		
	Number	Row %	*P*	Number	Row %	*P*
Total	5014	84		4218	61	
Recurrence score			<0.0001			0.13
Low (<18)	821	91		745	64	
Intermediate (18–30)	621	91		566	63	
High (>30)	165	83		137	66	
No oncotype	3407	81		2770	60	
Year diagnosed			0.06			0.001
2006–2007	1929	85		1639	58	
2008–2009	2097	83		1734	63	
2010	988	86		845	65	
Age at diagnosis, years			0.14			<0.001
24–39	361	81		293	51	
40–49	1621	83		1350	60	
50–59	2091	85		1784	63	
60–63	941	84		791	64	
Race-ethnicity			0.10			0.004
NH white	4263	84		3593	62	
NH black	188	79		148	49	
Hispanic	205	82		168	58	
Other/unknown	358	86		309	66	
Marital status			0.03			0.002
Not married	1427	82		1177	57	
Married	3507	85		2978	63	
Missing	80	79		63	63	
Prior cancer			0.77			0.24
No	4766	84		4011	61	
Yes	248	83		207	61	
State			0.04			0.14
CA	1985	83		1642	61	
GA	930	83		771	58	
KY	483	87		421	62	
NY	771	86		662	65	
OH	845	85		722	62	
Area			0.76			0.15
Rural	254	83		212	57	
Urban	4703	84		3959	62	
Missing	57	82		47	62	
Median household income			0.23			0.03
1 (lowest)	952			802	60	
2	921	84		766	61	
3	1071	83		892	58	
4	999	83		864	62	
5 (highest)	1014	86		847	66	
Missing	57	84		47	62	
Stage			0.001			0.44
I	3035	83		2504	61	
II	1979	87		1714	62	
Nodal involvement			0.001			0.50
N0	3767	83		3122	61	
N1mic	325	87		292	61	
N1	922	90		804	63	
HER2 status			0.66			0.35
Negative	2283	84		1928	62	
Borderline	55	87		48	52	
Unknown	2676	84		2242	61	
Hormone receptor status			<0.001			0.89
Both ER and PR positive	4299	85		3664	61	
Only ER or PR positive	715	77		554	61	
Histological grade			0.04			0.17
1–2	3737	85		3175	62	
3	1076	82		887	59	
Missing	201	78		156	58	
One-year comorbidities			0.17			0.08
0	4395	84		3709	62	
1 or more	619	82		509	58	
Surgery type			0.16			0.45
Breast-conserving surgery	3049	84		2569	62	
Mastectomy	1956	85		1642	61	
Unknown/missing	9	78		7	71	
Endocrine therapy						<0.001
Tamoxifen only	--			1512	58	
AIs only	--			1930	65	
Tamoxifen and AIs	--			776	58	
Adjuvant chemotherapy			<0.001			0.86
No	2585			2084	61	
Yes	2429			2134	61	

Nodal involvement: *N0* no cancer in the lymph nodes; *N1mic* lymph node cancer that can only be seen under a microscope (<2 mm in size); *N1* cancer at least 2 mm in size in at least one of three axillary lymph nodes. *NH* non-Hispanic, *HER2* human epidermal growth factor receptor 2, *ER* estrogen receptor, *PR* progesterone receptor, *AI* aromatase inhibitor

### Endocrine therapy initiation

We analyzed the relationship between GEP test results and endocrine therapy initiation in GEP-tested women (*N* = 1528): 10% of tested women did not initiate therapy. As seen in Table 2, after adjusting for all other variables, the RS was significantly associated with initiation (*P* = 0.02) in this model. As compared with women with low RS (91% of women), women with high RS results (83% of women) were significantly less likely to initiate endocrine therapy (OR 0.40 (95% CI 0.20 to 0.81); *P* = 0.01).

**Table 2:** Multivariable models of variables associated with endocrine initiation among women tested by Onco*type* Dx (*N* = 1528) and women who were eligible for testing by Onco*typ*e Dx (*N* = 4674)

	Onco*type* Dx-tested				Onco*type* Dx-eligible		
	OR		(95% CI)	*P*	OR	(95% CI)	*P*
Recurrence score				0.02			
Low (ref)							
Intermediate	0.98		(0.64–1.50)				
High	0.40		(0.20–0.81)				
Tested							<0.001
Yes					2.48	(2.03–3.04)	
No (ref)							
Year diagnosed				0.13			0.004
2006–2007 (ref)							
2008–2009	0.74		(0.49–1.13)		0.74	(0.61–0.89)	
2010–2012	1.17		0.65–2.09)		0.89	(0.70–1.15)	
Age at diagnosis				0.20			<0.001
24–39	0.49		(0.24–1.01)		0.59	(0.43–0.82)	
40–49	0.73		(0.48–1.11)		0.74	(0.60–0.90)	
50–59 (ref)							
60–63	0.80		(0.49–1.30)		1.01	(0.80–1.27)	
Race-ethnicity				0.06			0.19
NH white (ref)							
NH black	3.62		(0.83–15.81)		0.92	(0.61–1.38)	
Hispanic	0.62		(0.27–1.41)		0.89	(0.60–1.33)	
Other	2.52		(0.89–7.13)		1.44	(1.01–2.06)	
Marital status				0.16			0.12
Married (ref)							
Not married	0.76		(0.52–1.11)		0.87	(0.72–1.04)	
Prior cancer				0.65			0.84
No (ref)							
Yes	1.21		(0.52–2.80)		1.04	(0.71–1.53)	
State							0.004
CA (ref)							
GA	0.62		(0.32–1.20)		1.15	(0.85–1.55)	
KY	1.73		(0.72–4.14)		1.71	(1.19–2.47)	
NY	1.05		(0.50–2.19)		1.60	(1.16–2.20)	
OH	1.20		(0.58–2.50)		1.45	(1.05–2.00)	
Area				0.74			0.70
Rural (ref)							
Urban	1.17		(0.47–2.92)		1.08	(0.74–1.56)	
Median household income				0.24			0.07
1 (lowest (ref))							
2	1.23		(0.68–2.22)		0.96	(0.74–1.26)	
3	1.00		(0.57–1.76)		0.97	(0.74–1.26)	
4	1.84		(0.97–3.49)		1.35	(1.02–1.82)	
5 (highest)	1.10		0.59–2.07)		1.01	(0.75–1.36)	
Stage				0.14			0.52
I (ref)							
II	0.71		(0.45–1.12)		0.92	(0.71–1.19)	
Nodal involvement				0.22			0.10
N0	0.42		(0.14–1.26)		0.87	(0.64–1.17)	
N1mic	0.69		(0.18–2.62)		1.40	(0.91–2.17)	
N1 (ref)							
HER2 status				0.82			0.33
Negative	0.89		(0.49–1.62)		1.21	(0.55–2.92)	
Borderline	0.63		(0.13–3.06)		1.26	(0.94–1.56)	
Unknown (ref)							
Hormone receptor status							<0.001
ER and PR both positive (ref)							
Only ER or PR positive	0.80		(0.45–1.42)		0.58	(0.47–0.72)	
Histological grade				0.52			<0.001
1–2 (well/moderately differentiated (ref)							
3 (poorly or not differentiated)	1.18		(0.71–1.96)		0.68	(0.56–0.84)	
One year comorbidities				0.57			0.51
0 (ref)							
1 or more	0.86		(0.52–1.43)		0.92	(0.72–1.18)	
Surgery type				0.74			0.42
Breast-conserving surgery (ref)							
Mastectomy	0.94		(0.47–2.92)		0.93	(0.78–1.11)	
Chemotherapy				0.10			
No (ref)							
Yes	1.49		(0.92–2.39)				
Chemotherapy	Tested						<0.001
No	Yes	--			3.25	(2.53–4.16)	
	No	--					
Yes	Yes	--			1.35	(0.96–1.90)	
	No	--					

Nodal involvement: *N0* no cancer in the lymph nodes; *N1mic* lymph node cancer that can only be seen under a microscope (<2 mm in size); *N1* cancer at least 2 mm in size in at least one of three axillary lymph nodes. *ref* reference, *NH* non-Hispanic, *HER2* human epidermal growth factor receptor 2, *ER* estrogen receptor, *PR* progesterone receptor

**Fig. 1:**
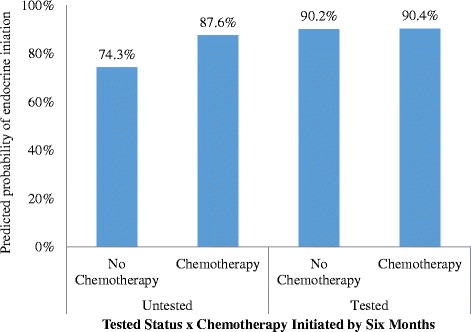
Adjusted odds of being tested was significantly higher for tested vs. untested women among women who did not initiate chemotherapy within six months of diagnosis. There were no significant differences in initiation by tested status in women had initiated chemotherapy within six months of diagnosis

Among all test-eligible women with complete data (*N* = 4674), 16% of women did not initiate therapy. Receipt of Onco*type* DX testing was associated with significantly greater likelihood of endocrine therapy initiation (10% vs. 19% non-initiation; OR 2.48 (95% CI 2.03 to 3.04); *P <*0.001). Younger age (*P <* 0.0001), being diagnosed after 2007 (*P =* 0.004), ER or PR positivity vs. ER and PR positivity (OR 0.58 (95% CI 0.47 to 0.72); *P <* 0.001), and poorly differentiated or undifferentiated tumor grade vs. well-differentiated or moderately differentiated grade (OR 0.68 (95% CI 0.56 to 0.84); *P* < 0.001) were associated with lower odds of initiation, while being outside our reference site of California (*P* < 0.004) was independently associated with greater odds of initiation of endocrine therapy. There also was a significant interaction between receipt of chemotherapy and tested status (*P* < 0.001). The adjusted odds of initiating endocrine therapy was significantly higher in tested vs. untested women among those women who did not receive adjuvant chemotherapy (OR 3.25 (95% CI 2.53 to 4.16)). There were no significant differences in initiation by tested status in women who had initiated chemotherapy within 6 months of diagnosis (OR 1.35 (95% CI 0.96 to 1.90)); Fig. 1).

### Discontinuation

**Fig. 2:**
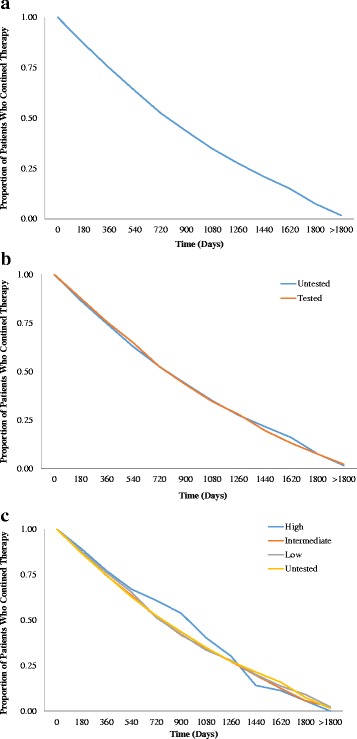
Kaplan-Meier curves for continuation of endocrine therapy among (**a**) all test-eligible women, (**b**) by tested status, and (**c**) by RS category

Discontinuation overall, by tested status and by RS category among tested women, is presented in Fig. 2. The proportion of patients who continued therapy during the study period was similar across tested vs. untested women and across RS category.

Among tested women, RS category was not associated with discontinuation. In the total cohort of eligible women (Table 3), tested status was not significantly related to discontinuation of endocrine therapy. Younger age (*P =* 0.002), being diagnosed after 2007 (*P <* 0.001), being non-Hispanic Black vs. white (hazard ratio (HR) 1.32 (95% CI 1.08 to 1.61)), unmarried vs. married (HR 1.11 (95% CI 1.02 to 1.21); *P =* 0.01) and having one or more comorbidities vs. none (HR 1.17 (95% CI 1.03 to 1.32); *P <* 0.01) were associated with greater odds of discontinuation. Being outside our reference site of California (*P* < 0.001), having higher household income (*P* < 0.001), and receiving aromatase inhibitors (AIs) (HR 0.77 (95% CI 0.70 to 0.87)) or a combination of AIs and tamoxifen (HR 0.76 (95% CI 0.68 to 0.85); *P <* 0.001) vs. tamoxifen alone were associated with completion of therapy within the time of our analysis.

**Table 3:** Multivariable model of variables associated with discontinuation of endocrine therapy among women who were eligible for Oncotype Dx testing (*N* = 3949)

	HR	(95% CI)	*P*
Tested			0.10
Yes	0.93	(0.85–1.02)	
No (ref)			
Year diagnosed			<0.001
2006–2007 (ref)			
2008–2009	1.62	(1.48–1.78)	
2010	2.71	(2.37–3.11)	
Age at diagnosis, years			0.002
24–39 (ref)			
40–49	0.75	(0.65–0.88)	
50–59	0.74	(0.63–0.88)	
60–63	0.80	(0.66–0.97)	
Race-ethnicity			0.03
NH white (ref)			
NH black	1.32	(1.08–1.61)	
Hispanic	1.09	(0.90–1.34)	
Other	0.93	(0.80–1.08)	
Marital status			0.01
Married (ref)			
Not married	1.11	(1.02–1.21)	
Prior cancer			0.87
No (ref)			
Yes	1.02	(0.85–1.22)	
State			<0.001
CA (ref)			
GA	1.01	(0.87–1.17)	
KY	0.81	(0.68–0.95)	
NY	0.77	(0.66–0.90)	
OH	0.86	(0.73–1.00)	
Area			0.36
Rural (ref)			
Urban	0.92	(0.77–1.10)	
Median household income			<0.001
1 (lowest (ref))			
2	0.99	(0.87–1.13)	
3	0.99	(0.87–1.12)	
4	0.93	(0.82–1.06)	
5 (highest)	0.76	(0.66–0.88)	
Stage			0.43
I (ref)			
II	1.05	(0.93–1.18)	
Nodal involvement			0.36
N0	1.09	(0.95–1.25)	
N1mic	0.97	(0.82–1.15)	
N1 (ref)			
HER2 status			0.61
Negative	0.95	(0.84–1.08)	
Borderline	1.08	(0.76–1.55)	
Unknown (ref)			
Hormone receptor status			0.69
ER and PR both positive (ref)			
Only ER or PR positive	1.02	(0.91–1.15)	
Histological grade			0.17
1–2 (well/moderately differentiated) (ref)			
3 (poorly or not differentiated)	1.07	(0.97–1.18)	
One year comorbidities			0.01
0 (ref)			
1 or more	1.17	(1.03–1.32)	
Surgery type			0.79
Breast-conserving surgery (ref)			
Mastectomy	1.01	(0.93–1.10)	
Endocrine therapy			<0.001
Tamoxifen only (ref)			
AIs only	0.77	(0.70–0.87)	
Tamoxifen and AIs	0.76	(0.68–0.85)	
Chemotherapy			0.82
Yes	1.01	(0.92–1.11)	
No (ref)			

Nodal involvement: *N0* no cancer in the lymph nodes; *N1mic* lymph node cancer that can only be seen under a microscope (<2 mm in size); *N1* cancer at least 2 mm in size in at least one of three axillary lymph nodes. *ref* reference, *NH* non-Hispanic, *HER2* human epidermal growth factor receptor 2, *ER* estrogen receptor, *PR* progesterone receptor, *AI* aromatase inhibitor

### Non-adherence

Our model of non-adherence among tested women suggested that RS category was not associated with non-adherence. In our model of non-adherence to endocrine therapy among test-eligible women (Table 4), tested status also was unrelated to adherence.

**Table 4:** Multivariable model of variables associated with adherence to endocrine therapy among women who were eligible for Onco*type* Dx testing (*N* = 3949)

	OR	(95% CI)	*P*
Tested			.09
Yes	0.88	(0.76–1.02)	
No (ref)			
Year diagnosed			0.003
2006–2007 (ref)			
2008–2009	0.81	(0.70–0.94)	
2010	0.74	(0.60–0.90)	
Age at diagnosis, years			0.047
24–39	1.50	(1.12–2.01)	
40–49	1.05	(0.88–1.25)	
50–59 (ref)			
60–63	1.00	(0.83–1.20)	
Race-ethnicity			0.06
NH white (ref)			
NH black	1.43	(1.01–2.03)	
Hispanic	1.04	(0.74–1.45)	
Other	0.80	(0.61–1.04)	
Marital status			0.003
Married (ref)			
Not married	1.24	(1.07–1.44)	
Prior cancer			0.30
No (ref)			
Yes	0.85	(0.62–1.16)	
State			0.17
CA (ref)			
GA	1.08	(0.85–1.39)	
KY	0.91	(0.69–1.21)	
NY	0.82	(0.64–1.05)	
OH	0.91	(0.71–1.18)	
Area			0.29
Rural (ref)			
Urban	0.85	(0.62–1.15)	
Median household income			0.20
1 (lowest (ref))			
2	0.98	(0.79–1.22)	
3	1.11	(0.90–1.37)	
4	0.98	(0.78–1.23)	
5 (highest)	0.86	(0.67–1.09)	
Stage			0.75
I (ref)			
II	0.97	(0.79–1.18)	
Nodal involvement			0.68
N0	1.07	(0.80–1.43)	
N1mic	1.11	(0.88–1.40)	
N1 (ref)			
HER2 status			0.49
Negative	1.03	(0.84–1.27)	
Borderline	1.45	(0.79–2.66)	
Unknown (ref)			
Hormone receptor status			0.96
ER and PR both positive (ref)			
Only ER or PR positive	0.99	(0.82–1.21)	
Histological grade			0.22
1–2 (well/moderately differentiated) (ref)			
3 (poorly or not differentiated)	1.11	(0.94–1.31)	
One-year comorbidities			0.006
0 (ref)			
1 or more	1.33	(1.09–1.62)	
Surgery type			0.97
Breast-conserving surgery (ref)			
Mastectomy	1.00	(0.87–1.15)	
Endocrine therapy			0.003
Tamoxifen only (ref)			
AIs only	0.77	(0.64–0.92)	
Tamoxifen and AIs	1.02	(0.85–1.24)	
Chemotherapy			0.31
Yes	0.92	(0.78–1.08)	
No (ref)			

Nodal involvement: *N0* no cancer in the lymph nodes; *N1mic* lymph node cancer that can only be seen under a microscope (<2 mm in size); *N1* cancer at least 2 mm in size in at least one of three axillary lymph nodes. *ref* reference, *NH* non-Hispanic, *HER2* human epidermal growth factor receptor 2, *ER* estrogen receptor, *PR* progesterone receptor, *AI* aromatase inhibitor

### Breast imaging

As seen in Table 5, RS was unrelated to our imaging outcomes.

**Table 5:** Multivariable model of variables associated with receipt of two or more breast imaging exams in the 6–26 months after diagnosis among tested women (*N* = 731)

	OR	(95% CI)	*P*
Recurrence score			0.06
Low or intermediate (ref)			
High	0.49	(0.23–1.03)	
Age at diagnosis, years			0.08
24–39	0.36	(0.14–0.90)	
40–49	1.18	(0.72–1.92)	
50–59 (ref)			
60–63	1.23	(0.69–2.19)	
Stage			0.48
I	1.22	(0.70–2.12)	
II (ref)			
Chemotherapy			0.33
Yes			
No (ref)	1.29	(0.77–2.15)	
Histological grade			0.84
1–2 (well/moderately differentiated) (ref)			
3 (poorly or not differentiated)	1.06	(0.58–1.95)	
Nodal involvement			0.58
N0 (ref)			
N1mic/N1	1.27	(0.54–2.99)	
Surgery type			<0.001
Breast-conserving surgery	3.75		
Unilateral mastectomy (ref)			

Nodal involvement: *N0* no cancer in the lymph nodes; *N1mic* lymph node cancer that can only be seen under a microscope (<2 mm in size); *N1* cancer at least 2 mm in size in at least one of three axillary lymph nodes

## Discussion

To our knowledge, this is the first study to assess endocrine therapy use and survivorship care among women who are eligible to receive Onco*type* Dx testing, a group that represents more than half of all new breast cancer cases each year. We found that multiple clinical, demographic and group-level variables, including the receipt of testing and test results, are associated with initiation of endocrine therapy in women under age 65 years with early-stage breast cancer, with additional variables associated with continuation and adherence.

Overall, non-initiation rates were comparable to the higher estimates of non-initiation seen in the literature [15–17]. Lower rates of initiation were observed among women having demographic or tumor characteristics associated with more aggressive disease (high RS and tumor grade, and younger age). While many of these patients with aggressive disease would have received chemotherapy, and this variable was included in our model, continuity of care for women receiving endocrine therapy following completion of chemotherapy could serve as a partial explanation [17, 29]. An additional disease-related explanation could be that some high-risk patients could have tumors with low ER positivity, defined as ER positivity between 1 and 10% [30, 31], given that prior guidelines did not recommend endocrine therapy in this group and there remains some uncertainty about the role of endocrine therapy in this setting [32, 33]. Receptor positivity is reported in local pathology reports and through the Onco*type* Dx test report, though we did not assess the concordance of these [34].

Patient’s misperceptions of the need for endocrine therapy or their interest in avoiding additional side effects of endocrine therapy following the receipt of chemotherapy could serve as an additional barrier to initiation. Some studies have found that women who receive chemotherapy have higher rates of initiation than chemotherapy-naïve women [17, 35], but others have found no such association [29, 36]. Our cohort and dataset differ in several ways from those in previous studies. The women in our cohort were younger (under age 65 years) than those in previous studies. Also, some studies have primarily used patient self-report data on the outcome of initiation and on receipt of chemotherapy. This could result in measurement differences that would impact the outcome across studies.

Our finding of non-initiation is of particular concern, given that the high RS is based on the assumption of receipt of endocrine therapy [12, 25]. Therefore, in the absence of endocrine therapy, this risk of recurrence may be even higher. Given the efficacy of endocrine therapy in all women with hormone-receptor-positive early-stage breast cancer, our results suggest that there is potential for improved adherence and reduced morbidity and mortality in those women with high RS, who comprise approximately 10% of tested women within this subgroup.

Further, among chemotherapy-naïve women, untested women were less likely to initiate endocrine therapy. This, in light of our findings of significantly higher initiation rates among tested vs. untested women, implies lower quality of care and non-adherence to clinical guidelines in the care of some women. Alternately, these differences in the tested vs. untested group may reflect a strong selection effect for testing, as we have previously reported that clinical markers were strongly associated with test use [10].

Unlike our findings on the initiation of endocrine therapy, disease with a high RS did not impact rates of discontinuation and nonadherence. We did find that discontinuation and nonadherence varied by several demographic and clinical factors, including age, race and comorbidity. Our study is consistent with previous findings showing that younger women were more likely to discontinue endocrine therapy than older women [18]. Numerous studies have identified variation in receipt of endocrine therapy by race and ethnicity [18, 37–39].

In addition, women who received only tamoxifen were less likely to continue and adhere than women on other regimens. Previous studies, most of which included an overall older cohort than that in the current study, demonstrate lower adherence among women who are on tamoxifen [18] or no effect [29, 36]. Our findings could reflect the comparatively younger age of our cohort. Side effects can limit women’s adherence and continuation of endocrine therapy and these side effects vary across women. Initial conversations about what side effects to expect [40] and ongoing communication between patients and their medical oncologists about side effects and options for switching may promote adherence [41].

While utilizing claims data comes with numerous strengths, it also comes with some limitations. These include the potential for coding inaccuracies, the need to censor patients based on their insurance coverage and presence in the dataset, and the overall generalizability of data outside of an insured USA population. Further, determination of recurrence or metastases using claims-based data is a known challenge [42]. Our study included a relatively small proportion of non-white and Hispanic participants. Our study also was limited by the high proportion of patients with unknown HER2 status, because most registries did not collect this element prior to 2010. We were unable to account for certain variables possibly related to survivorship care, such as the rates and severity of side effects from endocrine therapy, receipt of care in academic centers vs. community centers and other unmeasured patient-level variables.

Finally, our study may have limited generalizability to clinical practice in the USA because we were only able to include women under age 65 years with commercial health insurance in five USA states, but this also allowed us to contribute to a more specific understanding of breast cancer care among younger women. Our results reflect regional variation that might not reflect use in non-commercially insured women, uninsured women, and those in other states in the USA.

## Conclusion

The primary strength of our study is that it is among the first to assess the relationship between GEP testing and test results with breast cancer care in patients who are eligible for Onco*type* Dx testing. Our results suggest that in addition to previously reported associations between GEP testing and receipt of chemotherapy [14], there is an important association between GEP testing and the initiation of endocrine therapy. Additional studies should assess potential explanations for this variation and explore possible interventions to increase initiation of this efficacious therapy among all eligible women with hormone-receptor-positive breast cancer.

## Abbreviations

AI: aromatase inhibitor
ER: Estrogen receptor
GEP: Gene expression profiling
HER2: Human epidermal growth factor receptor 2
HIRD: HealthCore’s Integrated Research Database
HR: Hazard ratio
IHC: Immunohistochemical analysis
MRI: Magnetic resonance imaging
OR: Odds ratio
PR: Progesterone receptor
RS: Onco*type* DX® Breast Recurrence Score®
SEER: US National Cancer Institute’s Surveillance, Epidemiology, and End Results Program

## References

[1] American Cancer Society Cancer Facts & Figures 2016. Atlanta, GA: American Cancer Society. http://www.cancer.org/acs/groups/content/@research/documents/document/acspc-047079.pdf. Accessed 4 Nov 2016

[2] DeSantis C, Howlader N, Cronin KA, Jemal A (2011). Breast cancer incidence rates in U.S. women are no longer declining. Cancer Epidemiol Biomarkers Prev.

[3] Miller KD, Siegel RL, Lin CC, Mariotto AB, Kramer JL, Rowland JH (2016). Cancer treatment and survivorship statistics, 2016. CA Cancer J Clin.

[4] Rosenberg PS, Barker KA, Anderson WF. Estrogen receptor status and the future burden of invasive and in situ breast cancers in the United States. J Natl Cancer Inst. 2015;107(9). doi:10.1093/jnci/djv159. 10.1093/jnci/djv159 PMC4836802 26063794

[5] Ben-Baruch N, Hammerman A, Klang S, Liebermann N (2007). A prospective study of the impact of the 21-gene recurrence score assay on treatment decisions in N-, ER+ early stage breast cancer patients. 2007 ASCO Annual Meeting Proceedings (post-meeting edition). J Clin Oncol.

[6] Harris L, Fritsche H, Mennel R, Norton L, Ravdin P, Taube S (2007). American Society of Clinical Oncology 2007 update of recommendations for the use of tumor markers in breast cancer. J Clin Oncol.

[7] National Comprehensive Cancer Network NCCN Clinical Practice Guidelines in Oncology (NCCN Guidelines). Breast Cancer. Version 2.2016. 10.6004/jnccn.2016.0051 27059193

[8] Cress RD, Chen YS, Morris CR, Chew H, Kizer KW (2016). Underutilization of gene expression profiling for early-stage breast cancer in California. Cancer Causes Control.

[9] Hassett MJ, Silver SM, Hughes ME, Blayney DW, Edge SB, Herman JG (2012). Adoption of gene expression profile testing and association with use of chemotherapy among women with breast cancer. J Clin Oncol.

[10] O'Neill SC, Isaacs C, Chao C, Tsai HT, Liu C, Ekezue BF (2015). Adoption of gene expression profiling for breast cancer in US oncology practice for women younger than 65 years. J Natl Compr Canc Netw.

[11] Ravdin PM, Siminoff LA, Davis GJ, Mercer MB, Hewlett J, Gerson N (2001). Computer program to assist in making decisions about adjuvant therapy for women with early breast cancer. J Clin Oncol.

[12] Paik S, Tang G, Shak S, Kim C, Baker J, Kim W (2006). Gene expression and benefit of chemotherapy in women with node-negative, estrogen receptor-positive breast cancer. J Clin Oncol.

[13] Dinan MA, Mi X, Reed SD, Lyman GH, Curtis LH (2015). Association between use of the 21-gene recurrence score assay and receipt of chemotherapy among Medicare beneficiaries with early-stage breast cancer, 2005-2009. JAMA Oncol.

[14] Potosky AL, O'Neill SC, Isaacs C, Tsai HT, Chao C, Liu C (2015). Population-based study of the effect of gene expression profiling on adjuvant chemotherapy use in breast cancer patients under the age of 65 years. Cancer.

[15] Bowles EJ, Buist DS, Chubak J, Yu O, Johnson J, Chestnut J (2012). Endocrine therapy initiation from 2001 to 2008 varies by age at breast cancer diagnosis and tumor size. J Oncol Pract.

[16] Friese CR, Pini TM, Li Y, Abrahamse PH, Graff JJ, Hamilton AS (2013). Adjuvant endocrine therapy initiation and persistence in a diverse sample of patients with breast cancer. Breast Cancer Res Treat.

[17] Neugut AI, Hillyer GC, Kushi LH, Lamerato L, Leoce N, Nathanson SD (2012). Non-initiation of adjuvant hormonal therapy in women with hormone receptor-positive breast cancer: The Breast Cancer Quality of Care Study (BQUAL). Breast Cancer Res Treat.

[18] Hershman DL, Kushi LH, Shao T, Buono D, Kershenbaum A, Tsai WY (2010). Early discontinuation and nonadherence to adjuvant hormonal therapy in a cohort of 8,769 early-stage breast cancer patients. J Clin Oncol.

[19] Lin JH, Zhang SM, Manson JE (2011). Predicting adherence to tamoxifen for breast cancer adjuvant therapy and prevention. Cancer Prev Res (Phila).

[20] Murphy CC, Bartholomew LK, Carpentier MY, Bluethmann SM, Vernon SW (2012). Adherence to adjuvant hormonal therapy among breast cancer survivors in clinical practice: a systematic review. Breast Cancer Res Treat.

[21] Nekhlyudov L, Li L, Ross-Degnan D, Wagner AK (2011). Five-year patterns of adjuvant hormonal therapy use, persistence, and adherence among insured women with early-stage breast cancer. Breast Cancer Res Treat.

[22] Wirtz HS, Boudreau DM, Gralow JR, Barlow WE, Gray S, Bowles EJ (2014). Factors associated with long-term adherence to annual surveillance mammography among breast cancer survivors. Breast Cancer Res Treat.

[23] Gradishar W, Salerno KE (2016). NCCN Guidelines update: breast cancer. J Natl Compr Canc Netw.

[24] Khatcheressian JL, Hurley P, Bantug E, Esserman LJ, Grunfeld E, Halberg F (2013). Breast cancer follow-up and management after primary treatment: American Society of Clinical Oncology clinical practice guideline update. J Clin Oncol.

[25] Paik S, Shak S, Tang G, Kim C, Baker J, Cronin M (2004). A multigene assay to predict recurrence of tamoxifen-treated, node-negative breast cancer. N Engl J Med.

[26] American Joint Committee on Cancer (2010). AJCC Cancer Staging Manual.

[27] Elixhauser A, Steiner C, Harris DR, Coffey RM (1998). Comorbidity measures for use with administrative data. Med Care.

[28] Hess LM, Raebel MA, Conner DA, Malone DC (2006). Measurement of adherence in pharmacy administrative databases: a proposal for standard definitions and preferred measures. Ann Pharmacother.

[29] Hershman DL, Kushi LH, Hillyer GC, Coromilas E, Buono D, Lamerato L (2016). Psychosocial factors related to non-persistence with adjuvant endocrine therapy among women with breast cancer: the Breast Cancer Quality of Care Study (BQUAL). Breast Cancer Res Treat.

[30] Morgan DA, Refalo NA, Cheung KL (2011). Strength of ER-positivity in relation to survival in ER-positive breast cancer treated by adjuvant tamoxifen as sole systemic therapy. Breast.

[31] Prabhu JS, Korlimarla A, Desai K, Alexander A, Raghavan R, Anupama C (2014). A majority of low (1-10%) ER positive breast cancers behave like hormone receptor negative tumors. J Cancer.

[32] [no authors listed]. Tamoxifen for early breast cancer: an overview of the randomised trials. Early Breast Cancer Trialists' Collaborative Group. Lancet. 1998;351(9114):1451-1467 9605801

[33] Lin CM, Jaswal J, Vandenberg T, Tuck A, Brackstone M (2013). Weakly hormone receptor-positive breast cancer and use of adjuvant hormonal therapy. Curr Oncol.

[34] Badve SS, Baehner FL, Gray RP, Childs BH, Maddala T, Liu ML (2008). Estrogen- and progesterone-receptor status in ECOG 2197: comparison of immunohistochemistry by local and central laboratories and quantitative reverse transcription polymerase chain reaction by central laboratory. J Clin Oncol.

[35] Kimmick G, Anderson R, Camacho F, Bhosle M, Hwang W, Balkrishnan R (2009). Adjuvant hormonal therapy use among insured, low-income women with breast cancer. J Clin Oncol.

[36] Sheppard VB, Faul LA, Luta G, Clapp JD, Yung RL, Wang JH (2014). Frailty and adherence to adjuvant hormonal therapy in older women with breast cancer: CALGB protocol 369901. J Clin Oncol.

[37] Farias AJ, Du XL (2016). Ethnic differences in initiation and timing of adjuvant endocrine therapy among older women with hormone receptor-positive breast cancer enrolled in Medicare Part D. Med Oncol.

[38] Livaudais JC, Hershman DL, Habel L, Kushi L, Gomez SL, Li CI (2012). Racial/ethnic differences in initiation of adjuvant hormonal therapy among women with hormone receptor-positive breast cancer. Breast Cancer Res Treat.

[39] Roberts MC, Wheeler SB, Reeder-Hayes K (2015). Racial/ethnic and socioeconomic disparities in endocrine therapy adherence in breast cancer: a systematic review. Am J Public Health..

[40] Wuensch P, Hahne A, Haidinger R, Meissler K, Tenter B, Stoll C (2015). Discontinuation and non-adherence to endocrine therapy in breast cancer patients: is lack of communication the decisive factor?. J Cancer Res Clin Oncol.

[41] Bourmaud A, Rousset V, Regnier-Denois V, Collard O, Jacquin JP, Merrouche Y (2016). Improving adherence to adjuvant endocrine therapy in breast cancer through a therapeutic educational approach: a feasibility study. Oncol Nurs Forum.

[42] Chawla N, Yabroff KR, Mariotto A, McNeel TS, Schrag D, Warren JL (2014). Limited validity of diagnosis codes in Medicare claims for identifying cancer metastases and inferring stage. Ann Epidemiol.

